# Skeletal muscle dysregulation in rheumatoid arthritis: Metabolic and molecular markers in a rodent model and patients

**DOI:** 10.1371/journal.pone.0235702

**Published:** 2020-07-07

**Authors:** Nuria Casanova-Vallve, Dumitru Constantin-Teodosiu, Andrew Filer, Rowan S. Hardy, Paul L. Greenhaff, Victoria Chapman

**Affiliations:** 1 Division of Physiology, Pharmacology and Neuroscience, School of Life Science, University of Nottingham, Nottingham, England, United Kingdom; 2 Centre for Sports, Exercise and Osteoarthritis Research Versus Arthritis, University of Nottingham, Nottingham, England, United Kingdom; 3 MRC/Arthritis Research UK Centre for Musculoskeletal Ageing Research, University of Nottingham, Nottingham, England, United Kingdom; 4 Centre for Translational Inflammation Research, University of Birmingham, Birmingham, England, United Kingdom; 5 Institute of Metabolism and Systems Research, University of Birmingham, Birmingham, England, United Kingdom; 6 Pain Centre Versus Arthritis, University of Nottingham, Nottingham, England, United Kingdom; University of California, Davis, UNITED STATES

## Abstract

Rheumatoid arthritis (RA) is accompanied by pain, inflammation and muscle weakness. Skeletal muscle inflammation and inactivity are independently associated with muscle insulin resistance and atrophy. Our objective was to identify early molecular and biochemical markers in muscle from a rodent model of RA relative to control and subsequently identify commonality in muscle gene expression between this model and muscle from RA patients. Pain behaviour and locomotor activity were measured in a collagen-induced arthritis (CIA) model of RA (n = 9) and control (n = 9) rats. Energy substrates and metabolites, total alkaline-soluble protein:DNA ratio and mRNA abundance of 46 targeted genes were also determined in Extensor digitorum longus muscle. Expression of targeted mRNAs was quantified in Vastus Lateralis muscle from RA patients (n = 7) and healthy age-matched control volunteers (n = 6). CIA rats exhibited pain behaviour (p<0.01) and reduced activity (p<0.05) compared to controls. Muscle glycogen content was less (p<0.05) and muscle lactate content greater (p<0.01) in CIA rats. The bioinformatics analysis of muscle mRNA abundance differences from the control, predicted the activation of muscle protein metabolism and inhibition of muscle carbohydrate and fatty acid metabolism in CIA rats. Compared to age-matched control volunteers, RA patients exhibited altered muscle mRNA expression of 8 of the transcripts included as targets in the CIA model of RA. In conclusion, muscle energy metabolism and metabolic gene expression were altered in the CIA model, which was partly corroborated by targeted muscle mRNA measurements in RA patients. This research highlights the negative impact of RA on skeletal muscle metabolic homeostasis.

## Introduction

Rheumatoid arthritis (RA) is an autoimmune disease that causes joint inflammation and articular degradation following infiltration of inflammatory cells [1]. RA patients experience pain, which is a major contributor to reduced habitual physical activity levels and an increased sedentary lifestyle. Inflammation and physical inactivity have a negative effect on muscle mass and insulin sensitivity [2]. Animal models of RA, such as collagen-induced arthritis (CIA), have been used to investigate mechanisms of pain and joint pathology [3, 4], and the CIA model has been proven suitable to study rheumatoid cachexia [5], although mechanistic understanding is missing.

Overt inflammatory states are coupled to dysregulated muscle fuel metabolism and wasting, which in turn associate with changes in the molecular regulation of muscle mass and carbohydrate oxidation [6, 7]. Elevated levels of inflammatory cytokines have been implicated in aberrant phosphatidylinositol-3-kinase/Akt1 (PI3K/Akt1) signalling [8, 9], leading to net negative muscle protein balance and altered muscle metabolic flux [6, 7]. Similarly, chronic inflammation in RA has been linked to dysregulation of glucose and lipid metabolism in a murine model [10], but the underlying mechanisms are yet to be identified [11].

Studies exploring muscle function and metabolism in RA have reported increased muscle weakness and atrophy, particularly in type II muscle fibres [12, 13]. Muscle mass and function are dependent on the balance of muscle protein synthesis and breakdown. Reduced physical activity is associated with attenuated muscle protein synthesis, while inflammation not only attenuates muscle protein synthesis, but also increases protein breakdown [14]. Additionally, myogenic and neurotrophic factors, as well as their muscle receptors, are modulated by inflammation, which are differentially expressed under various physiological and pathological conditions [15].

In this study, we hypothesised that symptoms of pain and dysregulated muscle metabolism, arising directly from systemic and/or local RA-induced inflammation and/or indirectly as a consequence of reduced physical activity, would manifest in the CIA rat model. To test this hypothesis, pain behaviour, locomotor activity, systemic inflammation, muscle energy metabolism, targeted muscle gene expression, and markers of atrophy were measured in a control and a CIA rat model group. The translational relevance of differences from control in muscle gene expression in the CIA model was probed by comparing to muscle from RA patients with healthy age-matched volunteers.

## Materials and methods

All procedures in animals were approved by the University of Nottingham Ethical Review Committee and were performed under Home Office Project and Personal Licence authority (ASPA 1986) at the University of Nottingham. In accordance with UK Home Office regulations, 8 week old female Lewis rats (160–180 g, n = 18) were housed under standard conditions (12 h light/dark cycle), with unlimited access to food and water. After acclimation for 1 week, RA was induced which ensured age related motor or musculoskeletal decline was avoided in this study design. All outcome measurements were made by an observer blinded to the treatment applied. CIA and control animals were studied at pre-determined intervals for 18 days following injection with bovine collagen type II. A supplementary figure shows the experimental design in more detail (S1 Fig).

In RA patients, following local anaesthesia, needle biopsy samples were obtained from vastus lateralis muscle of patients with RA (n = 7, age 62.4±5 yrs) and age-matched healthy volunteers (n = 6, age 61.3±1.4 yrs). Patients had been diagnosed with RA for 2 to 35 yrs, and included patients prescribed anti-inflammatory medication (prednisolone, infliximab and methotrexate) (S1 Table). Patients gave informed consent, and archived RNA from muscle samples was provided by the University of Birmingham, which was analysed in tandem with archived muscle RNA from age-matched healthy volunteer stored in liquid nitrogen at the University of Nottingham.

Ethical approval for the RA patient and healthy volunteer procedures was provided by NRES Committee West Midlands (NHS REC reference: 07/H1204/191) and National Research Ethics Committee of the UK (13/WM/0075), respectively.

### Induction of RA in the rodent model

Rats were anaesthetised with isoflurane (2% in O_2_) prior to intradermal injections of bovine collagen type II for CIA rats (n = 9) or complete Freund’s adjuvant for control (n = 9), and randomly allocated as previously described [16]. Bovine collagen type II (4 mg/ml; MD Bioproducts) was dissolved in acetic acid 10 mM and emulsified 1:1 with complete Freund’s adjuvant (CFA), which was made with 1 mg *Mycobacterium Tuberculosis* in 1 ml of Incomplete Freund’s Adjuvant. Two hundred μl of bovine collagen type II emulsion (2 mg/ml) was injected in the base of the tail to induce arthritis (CIA model), and 200 μl of CFA emulsified 1:1 in acetic acid (10 mM) was injected in the control rats.

### Pain behaviour and locomotor activity

Mechanical paw withdrawal thresholds (PWT) were measured before and after model induction using calibrated Von Frey monofilaments as previously described [17, 18]. Von Frey monofilaments with bending forces from 2 g to 26 g in a logarithmic scale were applied to stimulate the plantar surface of the hindpaws. The lowest weight monofilament that elicited a withdrawal reflex was recorded as the PWT. Rats were habituated to PWT assessment for 2 days before the baseline measurement and tested at baseline, and on day 3, 7, 14 (n = 9 each group) and 18 (n = 7 each group; due to early development of paw swelling and severe inflammation) post-model induction/control.

The vertical and horizontal activity was quantified using locomotor activity boxes (LMA) as previously described [19]. Rats were housed in individual automated infrared activity boxes during the dark cycle. Beam-breaks were counted as intervals of 5 min time epochs for 30 min on the night before baseline, day 6, 13 (n = 9 each group), and before the end of the experiment (n = 7 each group).

### Indices of inflammation

Local inflammation was measured as paw swelling, thickness in millimetres, using digital electronic callipers (Mitutoyo, Andover, UK).

Tibiotalar joints were harvested, fixed in neutral buffered formalin and decalcified with Gooding and Stewart’s solution. Transversal sections were cut following the Osteoarthritis Research Society International (OARSI) guideline for ankyloses histological assessment. Three sections (8 μm) per rat from the tibiotalar joint at 60 μm intervals (n = 8 each group) were stained with H&E to examine synovia lining thickness and cellularity [20, 21]. The scoring was on a scale from 0 to 3, where: 0 = No cellularity and 1–2 synovial layers; 1 = Mild, no cellularity and 3–5 synovial layers; 2 = Moderate, mildly increased of cellularity and/or 6–8 synovial layers; 3 = Severe, cellularity and ≥ 9 synovial layers.

Tail vein blood (0.5 ml) was collected at the end of the experiment, and plasma was isolated. The cytokines interleukin-10 (IL-10), interleukin-6 (IL-6) and tumour necrosis factor-alpha (TNFα) were quantified using Quantikine ® ELISA kits (R&D Systems). Each cytokine was analysed in 75 μl of plasma according to the manufacturer’s instructions.

### Muscle metabolite measurements

Extensor digitorum longus (EDL) muscle from both hindlimbs was harvested under terminal anaesthesia (intraperitoneal injection of pentobarbital) and snap-frozen in liquid nitrogen. EDL muscle was freeze-dried and powdered. Muscle adenosine triphosphate (ATP), phosphocreatine (PCr), creatine, glycogen and lactate contents were determined utilising spectrophotometric assays as previously described [22].

### Muscle protein and DNA quantification

Freeze-dried powdered EDL muscle was used to quantify total alkaline-soluble protein and DNA contents as previously described [23]. The ratio protein:DNA is a reliable marker for muscle mass [24].

### mRNA extraction and quantitative PCR

Total mRNA was extracted from 20–30 mg of frozen wet EDL muscle from which the first-strand cDNA was synthesised and amplified using qPCR. Forty-six genes were selected based on their involvement in the regulation of skeletal muscle metabolic fuel use and protein metabolism, inflammation and neurotrophic events (S2 Table), and quantified using TaqMan low-density arrays (TLDA; Applied Biosystems).

Eighteen relevant genes were also quantified in the muscle from patients with RA and age-matched healthy control volunteers using TaqMan probes (Life Technologies; S3 Table).

The relative quantification of gene expression (fold-change) between CIA rats and control, and between RA patients and healthy volunteers, was calculated using the 2^-ΔΔCt^ formula. Hydroxymethylbilane synthase (HMBS) was used as a housekeeping gene due to its stability in catabolic states, such as sepsis [6, 7].

### Statistical analysis

Data are presented as mean ± SEM and analysed by Prism V.7 (GraphPad, San Diego, California, USA). The analysis of the mean differences between control and CIA groups for PWT, locomotor activity and paw thickness was performed using two-way ANOVA with Bonferroni corrections. Student’s unpaired t-test was used to compare metabolites and the protein:DNA ratio in CIA and control. Mann-Whitney test was used to compare plasma cytokines, synovitis and individual mRNA expression levels. The group mean differences for the two-tailed test were considered statistically different when p<0.05. Gene transcript analysis and interpretation in the rodent study was achieved using Ingenuity Pathway Analysis (IPA) software (Redwood City, CA, USA). This involved online access to a comprehensive, manually curated database and use of algorithms to identify relationships and pathways relevant to changes observed in mRNA data sets uploaded to IPA. All the raw data can be found in the online repository figshare.

## Results

### CIA model: Pain behaviour, reduced locomotor activity and inflammation

**Fig 1:**
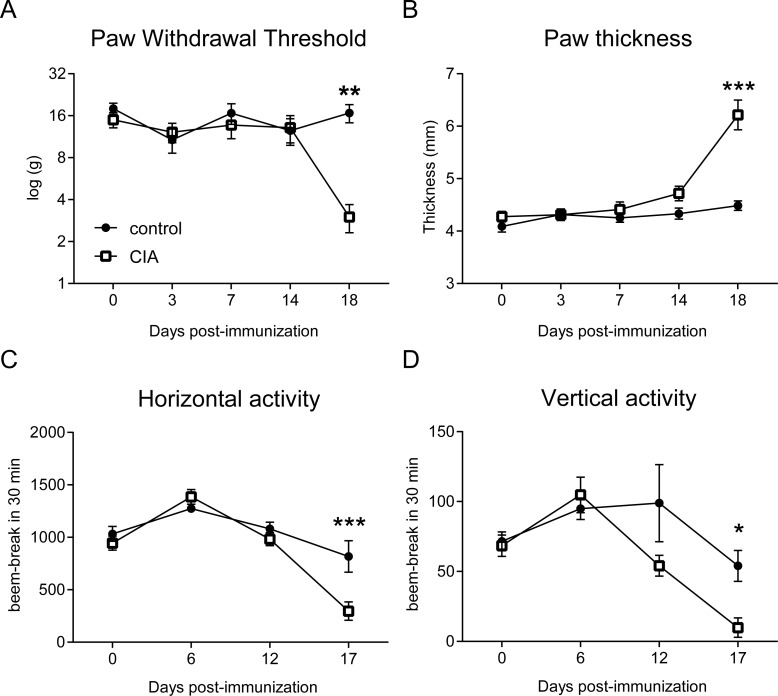
Increased pain and paw swelling accompanied by low locomotor activity in the rat CIA model. Paw withdrawal thresholds (A) were reduced, and paw thickness increased (B) 18 days after collagen-induced arthritis in CIA rats (n = 9) compared to control (n = 9). Both horizontal (C) and vertical (D) activity, which were measured during the dark cycle before testing pain, decreased on the night of day 17 post immunisation in CIA group compared to control. Results represent mean±SEM. *p<0.05, **p<0.01, ***p<0.001, significantly different from control.

During the first 14 days of the experiment, PWT and locomotor activity were stable in the CIA and control CFA groups (Fig 1A–1D). At later time-points, the CIA group presented with significantly lower PWTs (Fig 1A), increased paw thickness (Fig 1B) and decreased vertical and horizontal activity (Fig 1C and 1D) compared to control.

**Fig 2:**
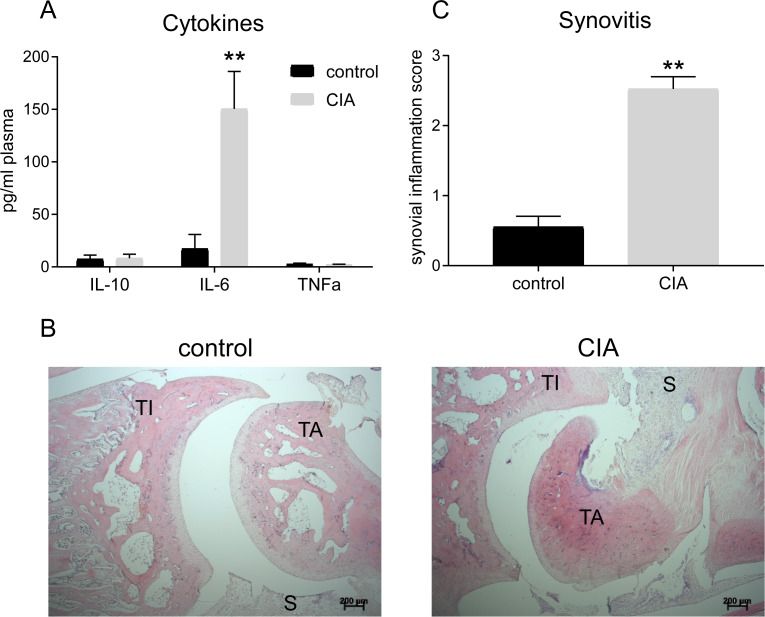
Indices of increased inflammation in the rat CIA model of RA. Plasma inflammatory cytokine IL-6 concentration (A) in the CIA group (n = 7) was greater than in control (n = 6) at the end of the study (day 18), but TNFα and IL-10 levels were similar to control. Histopathology (B), scale bar 200 μm, confirmed at the end of the study the development of arthritis in the tibiotalar joints of the CIA rats (n = 7) only, with increased cellularity, synovium lining thickness, and synovitis (C). TA- talus; TI- tibia; S- synovium. Results represent mean±SEM. **p<0.01, significantly different from control.

At the end of the study, plasma IL-10 and TNFα levels were comparable between groups, but IL-6 levels were markedly higher in the CIA group than in control (Fig 2A). H&E immunohistochemistry of the tibiotalar joint (ankle) confirmed an increase in the number of cells in the talus and synovium of CIA rats compared to control (Fig 2B). CIA rats exhibited significant ankle synovitis compared to control (Fig 2C).

### CIA model: Muscle glycogen and metabolite contents and alkaline-soluble protein to DNA ratio

**Fig 3:**
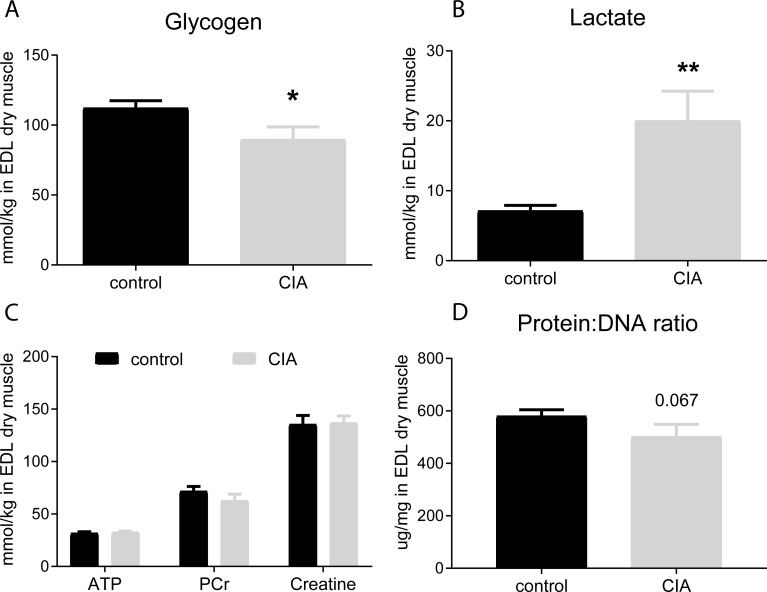
Muscle glycogen, intermediary metabolites and the protein: DNA ratio in the EDL muscle (n = 9 each group). Muscle glycogen content (A) was less and lactate (B) content greater in the EDL of CIA rats compared to control. In contrast, ATP, phosphocreatine (PCr) and creatine (Cr) content remained stable, indicating muscle viability in both groups (C). The alkaline-soluble protein: DNA ratio in CIA group tended to be lower than in control (D). Results represent mean±SEM. *p<0.05, **p<0.01, significantly different from control.

Muscle glycogen content was lower (Fig 3A) and lactate content greater (Fig 3B) in CIA rats compared to control. Muscle ATP, PCr and total creatine content were similar in the CIA and control groups, indicating comparable muscle viability and the absence of muscle necrosis (Fig 3C). Muscle protein:DNA ratio in the CIA group tended to be a lower than in control (p = 0.067, Fig 3D).

### Muscle mRNA expression

#### CIA model

**Fig 4:**
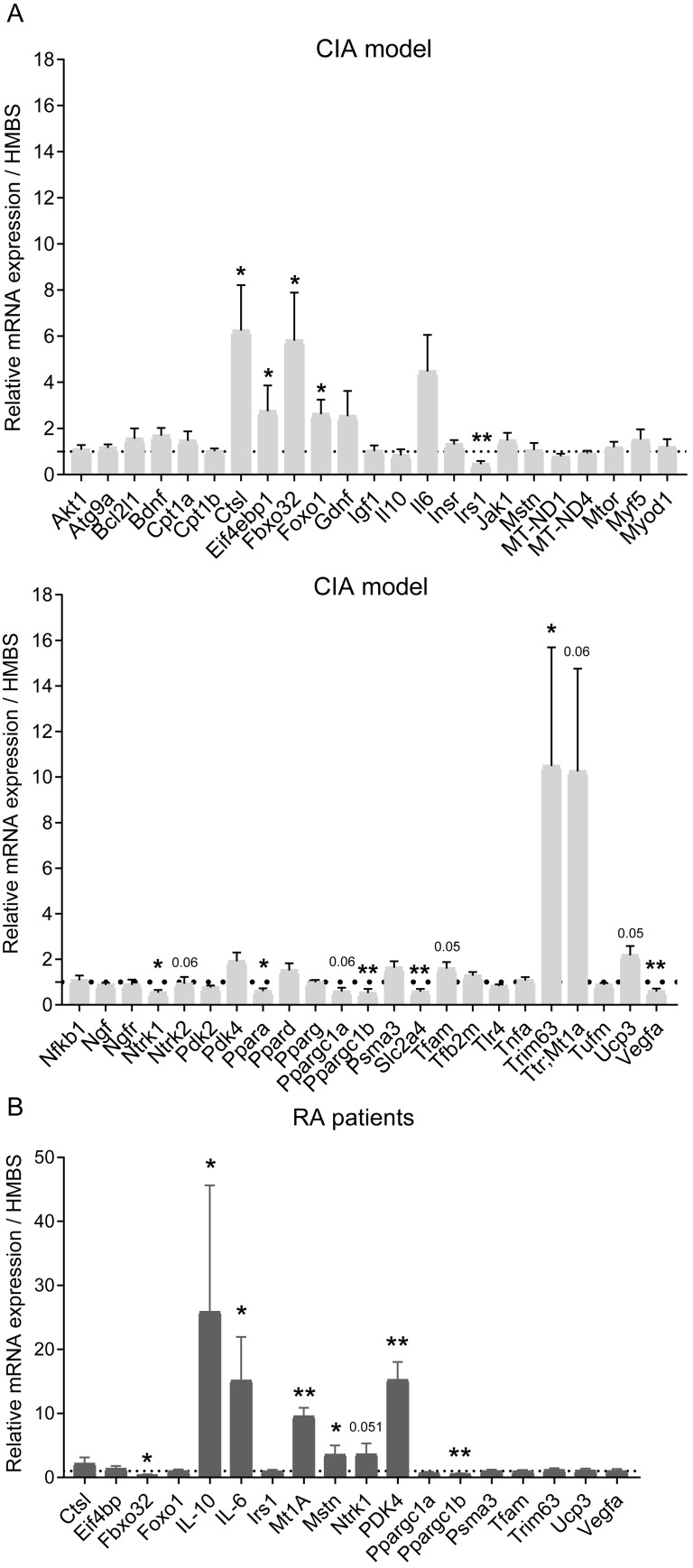
Expression of muscle mRNAs in the CIA model and patients with RA, relative to their corresponding control. Forty-six individual mRNAs in EDL muscle from CIA (n = 9) and control (n = 9) rats were quantified using TLDA cards (A). Eighteen of the most differentially regulated of these genes were also quantified in quadriceps muscle of patients with RA (n = 7) and expressed relative to the muscle from age-matched healthy control volunteers (n = 6; B). Results represent mean±SEM. *p<0.05, **p<0.01; significantly different from control.

Fig 4A shows the abundance of 46 targeted muscle mRNA transcripts quantified using TLDA cards, depicted as fold-change from control (set at 1, shown by the dotted line). The abundance of cathepsin-L (Ctsl), eukaryotic translation initiation factor 4E binding protein 1 (Eif4bp1), muscle atrophy f-box (Mafbx or Fbxo32), metallothionein 1A (Mt1a), muscle ring factor 1 (Murf1 or Trim63) and forkhead box protein1 (FoxO1) in the CIA group were deemed to be significantly higher than in control. Conversely, the mRNA abundance of insulin receptor substrate 1 (Irs1), peroxisome-proliferator activated receptor alpha (Ppara), peroxisome-proliferator activated receptor gamma coactivator 1 alpha (Ppargc1a), peroxisome-proliferator activated receptor gamma coactivator 1 beta (Ppargc1b), solute carrier family 2 member 4 (Slc2a4) and vascular endothelial growth factor alpha (Vegfa) were deemed to be significantly less in CIA than in control (Fig 4A).

#### Human rheumatoid arthritis patients

Eighteen mRNA transcripts were quantified in the patient and healthy volunteer muscle samples, of which eight were differentially regulated in the RA patients. mRNA expression of cytokines IL-10 and IL-6, MT1A, myostatin (MSTN) and pyruvate dehydrogenase kinase isoform 4 (PDK4) were markedly higher in RA patients than in control, (especially PDK4>10-fold; Fig 4B). Conversely, mRNA expression of MAFBX and PPARGC1β in RA patients were less than in healthy volunteers (Fig 4B).

### Metabolic predictions in the rodent CIA model

Upon uploading into Ingenuity Pathway Analysis (IPA) database, our analysed genes (fold change from control) were categorised into functional clusters by (IPA), and the network analysis was completed. Then, an IPA upstream regulator analysis (prediction) based on prior knowledge of expected effects between transcriptional regulators and their target genes stored in the Ingenuity®KnowledgeBase was requested. The analysis examined (1) how many known targets of each transcription regulator were present in our dataset, and (2) compared their direction of change (i.e. expression in the experimental samples relative to control) to what was expected from the annotated literature in order to predict likely relevant transcriptional regulators. If the observed direction of change was mostly consistent with a particular activation state of the transcriptional regulator (“activated” or “inhibited”), then a prediction was made about that activation state.

**Fig 5:**
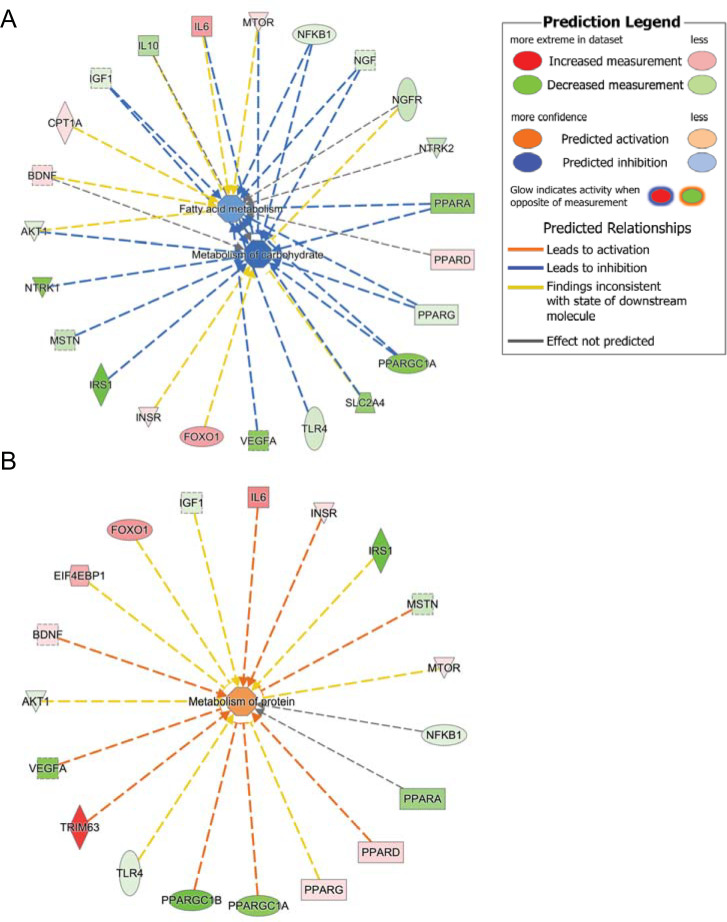
Schematic of the prediction of metabolic events in muscle of CIA rats by Ingenuity Pathway Analysis. The outer ring reflects the most differentially regulated mRNAs in CIA rats compared to control rats and with the colour intensity associated with a p-value and reflecting greater (red) or less (green) expression relative to control. The centre of each ring reflects Ingenuity Pathway Analysis prediction of inhibition of carbohydrate and fatty acid metabolism (A) and activation of protein metabolism (B) in the CIA group based on the collective mRNA expression changes from control.

Fig 5 depicts predicted cellular events in muscle from CIA relative to control. Based on the differential expression of 23 gene transcripts, IPA predicted the inhibition of carbohydrate (high confidence) and free fatty acid metabolism (medium confidence; Fig 5A). Of these mRNAs, the abundance of 16 in the CIA group was less than control: neurotrophic receptor tyrosine kinase 1 (NTRK1 or TRKA), IRS1, VEGFA, SLC2A4, PPARGC1A and PPARA. While the abundance of 7 mRNAs was greater than control, particularly IL-6 and FOXO1.

Fig 5B illustrates that 19 muscle gene transcripts were altered in abundance from control, from which IPA predicted the activation of protein metabolism (medium confidence). The abundance of eight mRNAs in the CIA group was greater than control, particularly MURF1 (TRIM63) while the abundance of 11 transcripts was less than control (VEGFα, PPARGC1β, PPARGC1α, PPARα and IRS1).

## Discussion

In the present study, the CIA model exhibited clinically relevant features of RA, including increased pain behaviour, paw swelling and reduced activity. Analysis of the hindlimb skeletal muscle of CIA rats provided new evidence that substantive changes in cellular muscle metabolism and gene expression occurred in tandem with these events but in the absence of overt muscle inflammation and necrosis. Based on the analysis of 46 targeted muscle gene transcripts, IPA predicted inhibition of carbohydrate and fatty acid metabolism, and activation of protein metabolism. In keeping with this, eight targeted genes associated with inflammation, muscle mass regulation, and muscle carbohydrate utilisation were differentially regulated in muscle from RA patients compared to healthy controls. However, the overlap in specific gene expression responses between the rat model and human patients was limited. Collectively, these findings highlight the impact of RA on skeletal muscle cellular and molecular regulation and pinpoint skeletal muscle as a potential target for intervention.

The increase in pain and reduced activity in CIA rats at the early stage of an acute inflammatory response is consistent with previous findings in this model [25] and adjuvant-induced arthritis (AIA) [26]. The presence of increased plasma IL-6 levels in conjunction with increased paw thickness and synovitis supports the contribution of a systemic and local inflammatory response. Pro-inflammatory cytokines, including IL-6, are known drivers of pain responses, including neuronal activity [27, 28] in several diseases, including RA [29]. Indeed, plasma IL-6 levels have been reported to be extremely high in pre-arthritis patients and the CIA model of RA, whereas levels of TNFα tend to decrease with the onset of disease [30, 31]. Despite the suggestive presence of systemic inflammation in the CIA model based on plasma IL-6 levels (Fig 2), muscle IL-6 mRNA expression was not significantly different from control. These data suggests either a temporal difference between systemic and inflammatory muscle responses occurred, or that systemic inflammation might have been the driver of muscle dysregulation in this model. In agreement with this latter contention, previous studies support the concept that skeletal muscle dysfunction in CIA rats is triggered by inflammation and cytokine release, rather than muscle disuse [32]. However, more studies are required to document the contributions of systemic and temporal muscle responses in the CIA model, and measuring plasma and muscle cytokine mRNA at different times of disease onset will help to understand them.

Muscle necrosis in the CIA model was not increased, evidenced by muscle ATP, PCr and free creatine contents being similar to control. Nevertheless, muscle glycogen content in the CIA model was less than control, while muscle lactate content was greater, reflecting dysregulation of carbohydrate metabolism as seen in other inflammatory states, and reported to occur via alterations in muscle Akt1/FOXO1 signalling [6, 7]. For example, in endotoxaemia, elevated muscle cytokines reportedly reduced muscle Akt1 phosphorylation, thereby dephosphorylating and increasing FOXO1 activity, resulting in the upregulation of FOXO1 downstream gene target MAFbx, MuRF1 and PDK4. Pyruvate dehydrogenase kinase 4 inhibits pyruvate dehydrogenase complex (PDC), which reduces mitochondrial pyruvate entry, thereby decreasing rates of muscle carbohydrate oxidation and increasing lactate production. This inefficient use of carbohydrate also results in more rapid utilisation of muscle glycogen stores. In short, the alterations in muscle intermediary metabolism in the CIA model are in keeping with muscle level events reported in inflammation [6, 33].

Similarly, genes regulating insulin sensitivity are known to be downregulated in muscle in inflammatory and catabolic states, including a mouse CIA model [10]. In our study, several genes linked to insulin signalling were downregulated, including Irs1, Slc2a4, Pparα, Ppargc1α and Ppargc1β. Similarly, lower expression of VEGFα was observed, which is associated with FOXO1 mediated suppression of metabolic flux, mitochondrial respiration and fatty acid synthesis [34]. Muscle Ntrk1 (TrkA) was downregulated in CIA rats. However, increased Ntrk1 is associated with myofibre regeneration in rat myogenic cells and pain in rat dorsal root ganglia in OA models [35, 36]. Based on the collective change from control in 23 muscle mRNA transcripts, IPA predicted inhibition of muscle carbohydrate metabolism with high confidence in the CIA model (Fig 4A).

Given that RA is a disease characterised by heightened autoimmune and inflammatory responses, we hypothesised that muscle mass would be reduced in this model. It has been reported that proteolytic pathways are upregulated in inflammatory and wasting states by changes in Akt1/FOXO1 signalling downstream targets, which include cathepsin-L and the muscle specific UPP ligases Mafbx and MuRF1 [37, 38]. Indeed, expression levels of proteolytic genes are increased in muscle from animal models of arthritis [39], including the ubiquitin-proteasome and autophagic-lysosomal proteolytic pathways [12]. Conversely, pharmacological dampening of muscle inflammation has been reported to preserve muscle mass by reversing inflammation-induced upregulation of these proteolytic pathways [33, 40]. It was, therefore, reassuring that expression levels of mRNAs involved in muscle protein breakdown, such as cathepsin-L, Mafbx (Fbxo32), Mt1A, Murf1 (Trim63) and FOXO1, were increased in the CIA model compared to control. Of note, IPA analysis revealed that 19 muscle gene targets were differentially regulated in the CIA model compared to control, which collectively predicted the activation of protein metabolism. In keeping with this, the muscle alkaline-soluble protein:DNA ratio showed a strong trend to be less in CIA compared to control.

In this study, mRNA abundance of eight genes associated with inflammation, muscle mass regulation and muscle carbohydrate utilisation were differentially regulated from control in muscle from RA patients. The expression of IL-10 and IL-6 in RA was substantially higher than in healthy control volunteers. Similarly, Mt1A was upregulated circa 10-fold from control. This gene is associated with oxidative stress [41] and is up-regulated in several models of muscle atrophy [38], particularly in the presence of elevated inflammation [42]. The marked elevation of muscle PDK4 mRNA over control in RA patients is in keeping with muscle inflammatory states and the impairment of carbohydrate oxidation [6, 7, 33, 40]. Interestingly, muscle myostatin mRNA expression was increased in RA patients, which has been observed in inflammation linked to human muscle wasting [7].

With the exception of muscle PPARGC1β, there was no evidence of an overlapping response in individual mRNAs when comparing the CIA model and RA patients. This could be attributable to a number of factors, including the significantly different muscle fibre composition of rat EDL vs human VL (the former is predominantly a fast-twitch fibred muscle [43], and the latter mixed-fibred [44]), and differences in metabolic stability between rodents and humans [45]. Another important consideration is the CIA model duration (~18 days), which, due to the severity of the model, had to be limited to ensure the welfare of the rats. By contrast, the RA patients had a long-term diagnosis and history of the disease (range 2–35 yrs), along with associated chronic medication (S1 Table), which likely impacted upon the comparisons between the CIA model and clinical disease. Another limitation of the study is the lack of further clinical information from RA patients, which could potentially facilitate the interpretation of findings and comparison with the CIA model. Nevertheless, it is clear that perturbations in skeletal muscle mass and fuel metabolism are linked to inflammation, and this was evident in both CIA model and RA patients, highlighting skeletal muscle as a site of dysregulation in RA, and a potential target for intervention.

## Conclusions

In conclusion, the CIA model of RA showed a systemic inflammatory response with the development of disease pathology reflected by pain, low locomotor activity, paw swelling and synovitis. These events were paralleled by muscle cellular and molecular events in CIA rats in keeping with the dysregulation of muscle fuel and protein metabolism, evidenced by lower muscle glycogen and greater muscle lactate contents, a tendency towards increased muscle atrophy, and widespread differences in the abundance of targeted mRNAs from control. There was, however, no evidence of muscle necrosis in the CIA model. Compared to the rodent model, RA patients showed more marked muscle inflammation, but also differential mRNA expression consistent with the dysregulation of muscle mass and fuel metabolism.

## Supporting information

S1 Fig Experimental design of the CIA study. (TIF)

S1 Table Clinical features from patients with rheumatoid arthritis. (DOCX)

S2 Table Genes included in the TaqMan low-density array with its catalogue number for rat. (DOCX)

S3 Table Genes selected to be quantified by TaqMan probes in RA patients. (DOCX)
