# Supplementary figures and images for: Skeletal muscle dysregulation in rheumatoid arthritis: Metabolic and molecular markers in a rodent model and patients
Source: PLoS One. 2020 Jul 7;15(7):e0235702. doi: 10.1371/journal.pone.0235702 (PMC7340297; doi:10.1371/journal.pone.0235702)

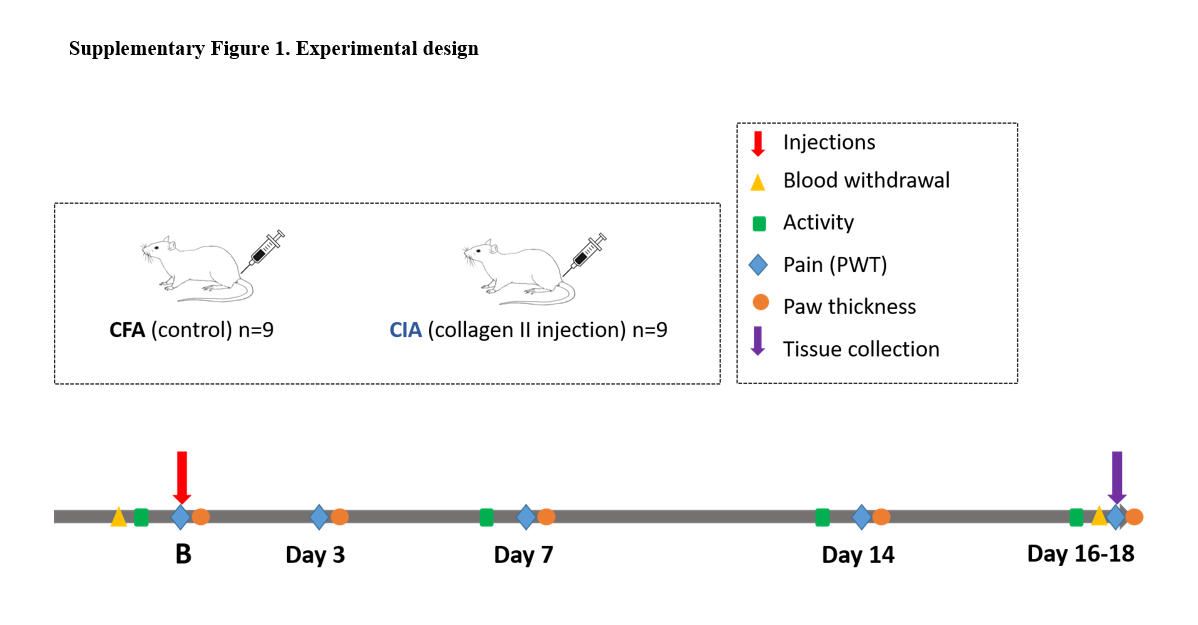

Supplement: S1 Fig — (TIF) [file pone.0235702.s001.tif]
