# Supplementary material for: Skeletal muscle dysregulation in rheumatoid arthritis: Metabolic and molecular markers in a rodent model and patients
Source: PLoS One. 2020 Jul 7;15(7):e0235702. doi: 10.1371/journal.pone.0235702 (PMC7340297; doi:10.1371/journal.pone.0235702)
Supplement: S1 Table — (DOCX) [file pone.0235702.s002.docx]

**S1 Table. Clinical features from patients with rheumatoid arthritis.**

| Patients | Age | Duration | Protein C-reactive (CRP) | Medication |
| --- | --- | --- | --- | --- |
| JRP001 | 62 | - | - | - |
| JRP006 | 63 | 35 years | 11 mg/L | Prednisolone |
| JRP036 | 67 | >20 years | 15 mg/L | Infliximab |
| JRP039 | 67 | >10 years | 25 mg/L | Methotrexate |
| JRP059 | 81 | 2 years | <3 mg/L | None |
| JRP083 | 60 | - | N/A | Unknown |
| JRP084 | 37 | - | <3 mg/L | Methotrexate |
