# Supplementary material for: Skeletal muscle dysregulation in rheumatoid arthritis: Metabolic and molecular markers in a rodent model and patients
Source: PLoS One. 2020 Jul 7;15(7):e0235702. doi: 10.1371/journal.pone.0235702 (PMC7340297; doi:10.1371/journal.pone.0235702)
Supplement: S2 Table — (DOCX) [file pone.0235702.s003.docx]

**S2 Table: Genes included in the Taqman low-density array with its catalogue number for rat.**

| Genes | Name | Catalogue number | |  |
| --- | --- | --- | --- | --- |
| *Mitochondria* |  | |  |  |
| Mtnd1 | Mitochondrial subunit ND1 | | Rn03296764_s1 |  |
| Ucp3 | Mitochondrial uncoupling protein 3 | | Rn00565874_m1 |  |
| Tfam | Transcription factor A mitochondrial | | Rn00580051_m1 |  |
| Tufm | Tu translational elongation factor mitochondrial | | Rn01509017_g1 |  |
| Tf2bm | Transcription factor B2 mitochondrial | | Rn01412502_m1 |  |
| Mtnd4 | Mitochondrial subunit ND4 | | Rn03296781_s1 |  |
| Bcl2l1 | Bcl-2-like 1 | | Rn00437783_m1 |  |
| *Angiogenesis* |  | |  |  |
| Vegfa | Vascular endothelial growth factor A | | Rn01511602_m1 |  |
| *Carbohydrate metabolism* | | | |  |
| Pdk2 | Pyruvate dehydrogenase kinase 2 | | Mm00446681_m1 |  |
| Slc2a4 (Glut4) | Glucose transporter 2 | | Rn00562597_m1 |  |
| Pdk4 | Pyruvate dehydrogenase kinase 2 | | Rn00585577_m1 |  |
| Foxo1 | Forkhead box protein 1 | | Rn01494868_m1 |  |
| *Lipid metabolism* | | | |  |
| Cpt1a | Carnitine palmitoyltransferase 1A | | Rn00682395_m1 |  |
| Cpt1b | Carnitine palmitoyltrasnferase 1B | | Rn00580702_m1 |  |
| *Insulin resistance* | | | |  |
| Igf1 | Insulin-like growth factor 1 | | Rn00710306_m1 |  |
| Insr | Insulin receptor | | Rn00690703_m1 |  |
| Irs1 | Insulin receptor substrate 1 | | Rn02132493_s1 |  |
| Ppara | Peroxisome proliferator-activated receptor alpha | | Rn00566193_m1 |  |
| Ppard | Peroxisome proliferator-activated receptor delta | | Rn00565707_m1 |  |
| Pparg | Peroxisome proliferator-activated receptor gamma | | Rn00440945_m1 |  |
| Ppargc1a | Peroxisome proliferator-activated receptor gamma coactivator 1 alpha | | Rn00580241_m1 |  |
| Ppargc1b | Peroxisome proliferator-activated receptor gamma coactivator 1 beta | | Rn00598552_m1 |  |
| *Endogenous genes* | | | |  |
| Hmbs | Hydroxymethylbilane synthase | | Rn01421881_m1 |  |
| 18S (Hs) | 18 S ribosomal RNA | | Mm04277571_s1 |  |
| *Myogenic factors* | | | |  |
| Myf5 | Myogenic factor 5 | | Rn01502778_m1 |  |
| Myod1 | Myogenic differentiation 1 | | Rn00598571_m1 |  |
| Mstn | Myostatin | | Rn00569683_m1 |  |
| *Protein synthesis* | | |  |  |
| Eif4ebp1 | Eukaryotic translation initiation factor 4E binding protein 1 | | Rn00587824_m1 |  |
| Mtor | Mammalian target of rapamycin | | Rn00693900_m1 |  |
| Murf1 | Muscle RING-finger protein 1 | | Rn00590197_m1 |  |
| Mafbx | Muscle atrophy f-box | | Rn00591730_m1 |  |
| Psma3 | Proteasome subunit alpha type 3 | | Rn02587201_s1 |  |
| Mt1A | Metallothionein 1A | | Rn01536930_g1 |  |
| Ctsl | Cathepsin L | | Rn04341361_m1 |  |
| Akt1 | Serine/threonine-protein kinase 1 | | Rn00583646_m1 |  |
| Atg9 | Autophagy-related 9 | | Rn01400691_m1 |  |
| *Inflammation* | | | |  |
| Nfkb1 | Nuclear factor kappa-light-chain-enhancer of activated B cells | | Rn01399572_m1 |  |
| Il6 | Interleukin 6 | | Rn01410330_m1 |  |
| Il10 | Interleukin 10 | | Rn01644839_m1 |  |
| Tlr4 | Toll-like receptor 4 | | Rn00569848_m1 |  |
| Jak1 | Janus kinase 1 | | Rn01763899_m1 |  |
| Tnfa | Tumour necrosis factor alpha | | Rn99999017_m1 |  |
| *Neurotrophic factors* | | | |  |
| Ngf | Nerve growth factor | | Rn01533872_m1 |  |
| Bdnf | Brain-derived neurotrophic factor | | Rn01484928_m1 |  |
| Gdnf | Glial cell line-derived neurotrophic factor | | Rn01402432_m1 |  |
| Trka | Tropomyosin receptor kinase A | | Rn00572130_m1 |  |
| Trkb | Tropomyosin receptor kinase B | | Rn01441749_m1 |  |
| p75 | Low-affinity nerve growth factor receptor | | Rn00561634_m1 |  |
