# Supplementary material for: Skeletal muscle dysregulation in rheumatoid arthritis: Metabolic and molecular markers in a rodent model and patients
Source: PLoS One. 2020 Jul 7;15(7):e0235702. doi: 10.1371/journal.pone.0235702 (PMC7340297; doi:10.1371/journal.pone.0235702)
Supplement: S3 Table — (DOCX) [file pone.0235702.s004.docx]

**S3 Table: Genes selected to be quantified by Taqman probes in RA patients.**

| Gene | Catalogue number |
| --- | --- |
| PPARGC1A | Hs00173304_m1 |
| IL6 | Hs00174131_m1 |
| IRS1 | Hs00178563_m1 |
| FOXO1 | Hs00231106_m1 |
| PSMA3 | Hs00541061_m1 |
| EIF4EBP1 | Hs00607050_m1 |
| HMBS | Hs00609297_m1 |
| MURF1 | Hs00822397_m1 |
| MT1A | Hs00831826_s1 |
| VEGFA | Hs00900055_m1 |
| IL10 | Hs00961622_m1 |
| CTSL | Hs00964650_m1 |
| MSTN | Hs00976237_m1 |
| PPARGC1B | Hs00993805_m1 |
| NTRK1 | Hs01021011_m1 |
| PDK4 | Hs01037712_m1 |
| MAFBx | Hs01041408_m1 |
| TFAM | Hs01082775_m1 |
| UCP3 | Hs01106052_m1 |
